# Virtual Flood Experience Enhances Preparedness Through Emotional and Neural Engagement

**DOI:** 10.1111/risa.70407

**Published:** 2026-11-04

**Authors:** Toshio Fujimi, Takanori Kochiyama, Wataru Sato, Masahide Watanabe

**Affiliations:** ^1^ Disaster Prevention Research Institute Kyoto University Uji Kyoto Japan; ^2^ Brain Activity Imaging Center ATR‐Promotions Seika‐cho, Soraku‐gun Kyoto Japan; ^3^ Guardian Robot Project RIKEN Seika‐cho, Soraku‐gun Kyoto Japan; ^4^ Faculty of Economics Ryukoku University Fushimi‑ku Kyoto Japan

**Keywords:** climate adaptation, flood preparedness, functional magnetic resonance imaging (fMRI), risk communication, virtual reality

## Abstract

As climate change intensifies flood risks, strengthening individual preparedness is essential for sustainable societies. This study tested whether first‐person three‐dimensional computer graphics (3DCG) flood videos can complement official flood maps in motivating preparedness. We used these materials as operationalizations of experiential and descriptive information, respectively. In a preregistered web experiment with 1000 participants, those who viewed flood videos showed a small but statistically significant increase in preparedness intentions compared with those who viewed flood maps. Mediation analyses showed that, among the measured psychological variables, only fear had a significant indirect effect, suggesting that stimulus‐evoked fear helped explain this increase. We also conducted a functional magnetic resonance imaging (fMRI) study with 45 participants using a within‐subjects design. Flood videos elicited greater right amygdala activation than flood maps, consistent with fear‐related processing. Together, these findings provide convergent behavioral and neural evidence that first‐person 3DCG flood videos evoke stronger fear‐related responses than flood maps and that subjective fear helps account for their effect on preparedness intentions. The results suggest that visually vivid flood videos may complement map‐based communication by increasing emotional salience and promoting flood‐preparedness intentions.

## 1 Introduction

Floods cause tremendous economic damage and human casualties globally and are projected to increase in intensity in the future due to climate change. People living in flood‐prone areas are required to take flood preparedness actions at the individual and household levels because structural measures such as dams and dikes alone cannot provide complete protection from increasing flood threats (Kuhlicke et al. 2020). However, private flood preparedness actions have not been sufficiently implemented even though they effectively protect individuals and family lives and reduce flood damage (Kreibich et al. 2015; Meyer and Kunreuther 2017). Thus, promoting private flood preparedness remains a significant challenge for sustainable disaster risk reduction and climate adaptation.

Flood threats are usually communicated to residents through “descriptive information” (Weber 2006; Hertwig and Erev 2009) that conveys probabilities and consequences of flood using symbolic representations such as text, numbers, graphs, and maps: typically flood hazard or flood risk maps (Mudashiru et al. 2021). The descriptive information is processed with reasoning‐based appraisal that requires cognitive effort for interpretation (Loewenstein et al. 2001; Weber 2006; Hertwig and Erev 2009). Most residents face difficulty in interpreting descriptive flood information to properly imagine flooding situations because flooding is a rare event causing different situations from normal ones. Mental images of flood damage vary widely among individuals, with flood damage generally being underestimated by people who have never experienced it (Siegrist and Gutscher 2008; Mol et al. 2020). This suggests that descriptive information may fail to convey flood threats in a way that effectively promotes flood preparedness.

Emerging virtual reality (VR) technologies offer a promising opportunity to address this challenge by providing “experiential information” (Epstein 1994; Loewenstein et al. 2001; Leiserowitz 2006; Weber 2006). This study defines experiential information as information conveyed through perceptual representations, including images (Keller et al. 2006; Rickard et al. 2017; Lindner et al. 2019), sounds, and videos (Dootson et al. 2023; Wilhelmi et al. 2024). Experiential information can communicate flooding to residents in a more concrete and vivid manner than conventional flood maps. Rapidly growing VR technologies have drastically improved the quality of experiential information to expand their usage in flood risk communication (Bakhtiari et al. 2023). Experiential information may engage affective processing that operates rapidly and intuitively with minimal cognitive load, which can evoke appropriate emotion to act on flood preparedness (Brosch 2021). Experiential information generally promotes flood preparedness (Keller et al. 2006; Rickard et al. 2017; Lindner et al. 2019, Bernhardt et al. 2019; Mol et al. 2022), and some studies have inferred that emotional response evoked by experiential information is a key factor for promoting flood preparedness (Loewenstein et al. 2001; Leiserowitz 2006; Weber 2006).

Although experiential information may evoke emotional responses, it should be distinguished from fear appeal communication. Fear appeals are persuasive messages that use threat and fear arousal as part of a strategy to motivate protective action, often by emphasizing threat severity, susceptibility, and recommended responses (Witte and Allen 2000; Tannenbaum et al. 2015). In contrast, the experiential information examined in this study was designed to provide perceptually vivid representations of possible flooding situations based on official flood‐risk information, rather than to use fear arousal as an explicit persuasive strategy.

To understand why experiential and descriptive information may influence preparedness differently, we draw on dual‐process theories of judgment and decision‐making. These theories distinguish between a fast, automatic, affective, and associative mode of processing, often referred to as Type 1 processing, and a slower, deliberative, analytic, and cognitively effortful mode of processing, often referred to as Type 2 processing (Stanovich and West 2000; Evans and Stanovich 2013). Within this framework, experiential and descriptive information can be viewed as two communication formats that may differentially emphasize affective and analytic appraisal in risk communication (Loewenstein et al. 2001; Slovic et al. 2004; Weber 2006).

In parallel to the distinction between experiential and descriptive information, neural processing for responding to a threat can be roughly divided into two processes: survival (precognitive) and cognitive circuits (LeDoux and Pine 2016; Engelen and Mennella 2023). When mammals, including humans, face a threat, the survival circuit is automatically activated, which primarily involves subcortical areas, resulting in physiological and behavioral responses (Panksepp 2011; Adolphs 2013; Fanselow 2018). This evolutionarily old process can elicit emotion quickly without any cognitive effort (Tamietto and de Gelder 2010; Winkielman and Gogolushko 2018). Contrarily, individuals’ emotions can also be aroused through cognitive circuit processing with symbolic representations or language (LeDoux and Pine 2016; Barrett 2017). Higher order cortices may interpret them to evoke emotion with physiological and defensive reactions. Although these two circuits partially overlap and interact with each other, the former can elicit stronger emotions than the latter. Several studies have shown that images evoke stronger physiological responses (Larsen et al. 2003) and behaviors than words do (Winkielman and Gogolushko 2018).

Taken together, we hypothesized that experiential information is processed with different neural processes compared to descriptive information, which results in evoking stronger emotion to promote flood preparedness. Brain imaging methods, such as functional magnetic resonance imaging (fMRI), enable investigation of information processing inside the brain that cannot be accessed with conventional measures such as behavioral observations and self‐reports (Sawe and Chawla 2021; Doell et al. 2023). Examining these mechanisms in the context of climate adaptation contributes to understanding how different modes of communication influence disaster preparedness and offers insights for strategies that engage the public in building resilient, sustainable societies.

To investigate this hypothesis, we conducted a web experiment and fMRI experiment using virtual three‐dimensional computer graphic (3DCG) videos of floods as one operationalization of experiential information and official flood maps as one operationalization of descriptive information. The web experiment with a between‐subject design examined at a subjective level whether and how flood videos could increase subjective emotion and the intention of flood preparedness compared to flood maps. Then, the fMRI experiment with a within‐subject design investigated the effect of emotion at the neural level in response to the flood videos versus maps. By combining both experiment results, we examined the hypothesis that compared to descriptive information, experiential information activates subjective and neural emotion to increase the intention of flood preparedness.

## 2 Web Experiment

First, we investigated the research hypothesis at a subjective level. Previous studies have indicated that experiential information about flood threats increased negative emotions such as fear and worry (Spence et al. 2011; Bubeck et al. 2012) and risk perceptions (on frequency and magnitude) (Rickard et al. 2017; Mol et al. 2022; Dootson et al. 2023), which are often positively related to the intention of flood preparedness (Bubeck et al. 2012; van Valkengoed and Steg 2019; Noll et al. 2022). In addition, personal relevance was included to capture the extent to which participants perceived flood risk as relevant to themselves (Demuth 2018). Thus, we focused on fear, worry, risk perception, and personal relevance as possible factors to affect the intention of flood preparedness. In particular, fear deserves special attention because it is closely connected to visceral responses (Adolphs 2013; Fanselow 2018), which may in turn influence worry, risk perception, and personal relevance, whereas the reverse influence from these variables to fear is less theoretically expected. Based on these findings, we investigated differences in the relationships among fear, worry, risk perception, personal relevance, and intention of flood preparedness when participants were presented with experiential information compared to when they were presented with descriptive information.

### 2.1 Methods

We conducted a web‐based survey experiment with 1000 residents of Osaka City, a flood‐prone megacity in Japan, from December 16 to 18, 2024. The study protocol was approved by the Ryukoku University Institutional Review Board (approval no. 2024‐52). The preregistration of this study is available on the Open Science Framework (OSF: https://osf.io/2sw8a).

#### 2.1.1 Participants

The web experiment recruited 1000 participants from a panel managed by Cross Marketing Inc., one of Japan's largest Internet research companies, which had 10.9 million registered members as of July 2024. Invitations were randomly distributed to panel members, and responses were accepted on a first‐come, first‐served basis. Participants were informed that the survey would take approximately 20–30 min to complete and that they could withdraw at any time.

#### 2.1.2 Sample Size

The sample size was determined based on a power analysis to test the hypothesis that participants’ intention to undertake flood preparedness would increase after exposure to flood videos compared with flood maps. A pretest with 200 residents of Osaka City, conducted in September 2024, indicated an effect size of d=0.29. Using G*Power 3.1.9.7, we calculated that a sample size of 376–620 would be required to detect this effect size with a two‐tailed test at α=0.05 and power (1−β)=0.80−0.95. Within this range, we selected 500 participants for the two experimental conditions (flood videos and flood maps). An equal number of participants was assigned to the two control conditions (normal videos and normal maps), yielding a total sample size of 1000 for the web experiment.

#### 2.1.3 Design and Stimuli

The experiment employed a between‐subjects 2 × 2 factorial design with two factors: media (video vs. map) and situation (flood vs. normal). Participants were randomly assigned to one of four conditions: flood videos (*N* = 249), flood maps (*N* = 256), normal videos (*N* = 246), and normal maps (*N* = 249), as illustrated in Figure 1.

**FIGURE 1:**
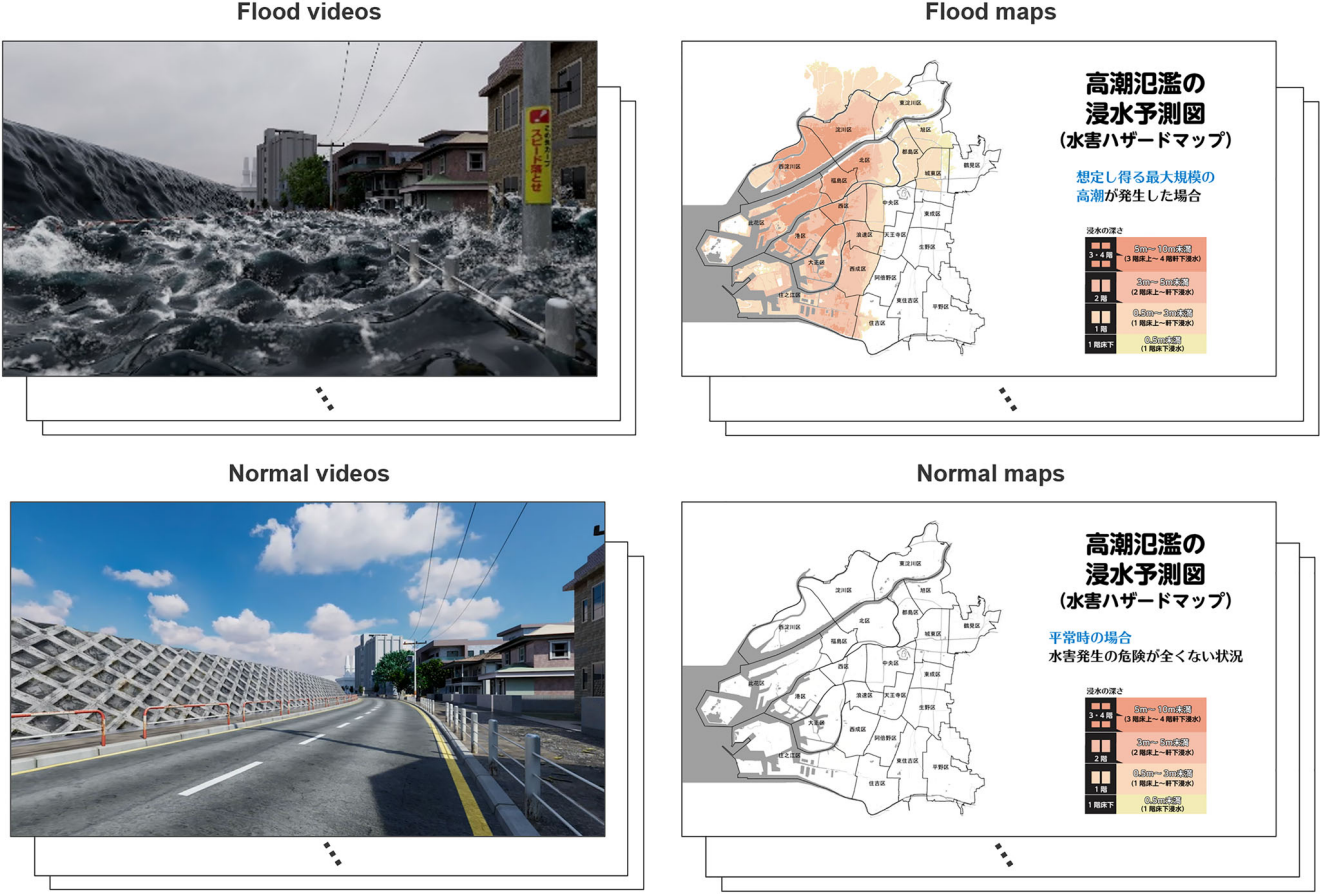
Examples of experimental stimuli. There were four experimental conditions: “flood videos,” “flood maps,” “normal videos,” and “normal maps.” Each condition includes nine different stimuli. The full set of stimuli is provided in OSF: https://osf.io/2sw8a.

The flood video condition included nine different videos depicting flooding scenarios from a first‐person perspective on the streets of Osaka City. Each video was 16 s long and presented without sound. With input from seven experts (two meteorologists, two hydrologists, two coastal engineers, and one landscape engineer), the videos were designed to represent predicted flooding situations shown in official flood hazard maps provided by the local government. The normal videos served as control stimuli for the flood videos. Each normal video, also 16 s long and without sound, depicted a sunny‐day scene from the same first‐person viewpoints as the corresponding flood videos.

The flood map condition comprised nine different maps illustrating flood hazards in Osaka City. Five maps indicated predicted inundation depths: one for storm surge in Osaka Bay and four for flooding in the city's major rivers. The remaining four maps depicted areas facing imminent danger during a heavy rainstorm on June 2, 2023, including inland flooding, river flooding, heavy rainfall, and an issued flood warning. All maps were provided to residents by the local government or the Japan Meteorological Agency via the web. The normal maps served as control stimuli for the flood maps. Each normal map was identical to its corresponding flood map, except that all flood‐related information—such as color coding for inundation depth—was removed. The full set of stimuli is provided in OSF: https://osf.io/2sw8a

All stimuli were presented without sound. This visual‐only presentation was chosen because flood maps are inherently visual stimuli; adding sound only to the video condition would have introduced an additional auditory component and reduced comparability between the two communication formats.

#### 2.1.4 Experimental Procedure

The procedure of the web experiment is illustrated in Figure 2. At the outset, participants were asked about their current worry, risk perceptions (frequency and magnitude), perceived personal relevance of floods, and both their prior actions and intentions regarding flood preparedness. They were then randomly assigned to one of four experimental conditions: flood videos, normal videos, flood maps, or normal maps. Each participant was exposed to nine stimuli from the assigned condition in random order. Immediately after each 16‐s stimulus, participants rated their fear on a four‐point scale (1 = not fearful, 4 = very fearful). After completing all nine stimuli, participants were again asked about their worry, risk perceptions (frequency and magnitude), perceived personal relevance of floods, and flood preparedness intentions. Finally, demographic information was collected, including age, gender, education, family size, housing type, flood evacuation experience, and flood damage experience. Table A1 provides a list of all variables in this study. Random assignment produced well‐balanced groups with respect to demographic and psychological variables (Tables A2 and A3).

**FIGURE 2:**
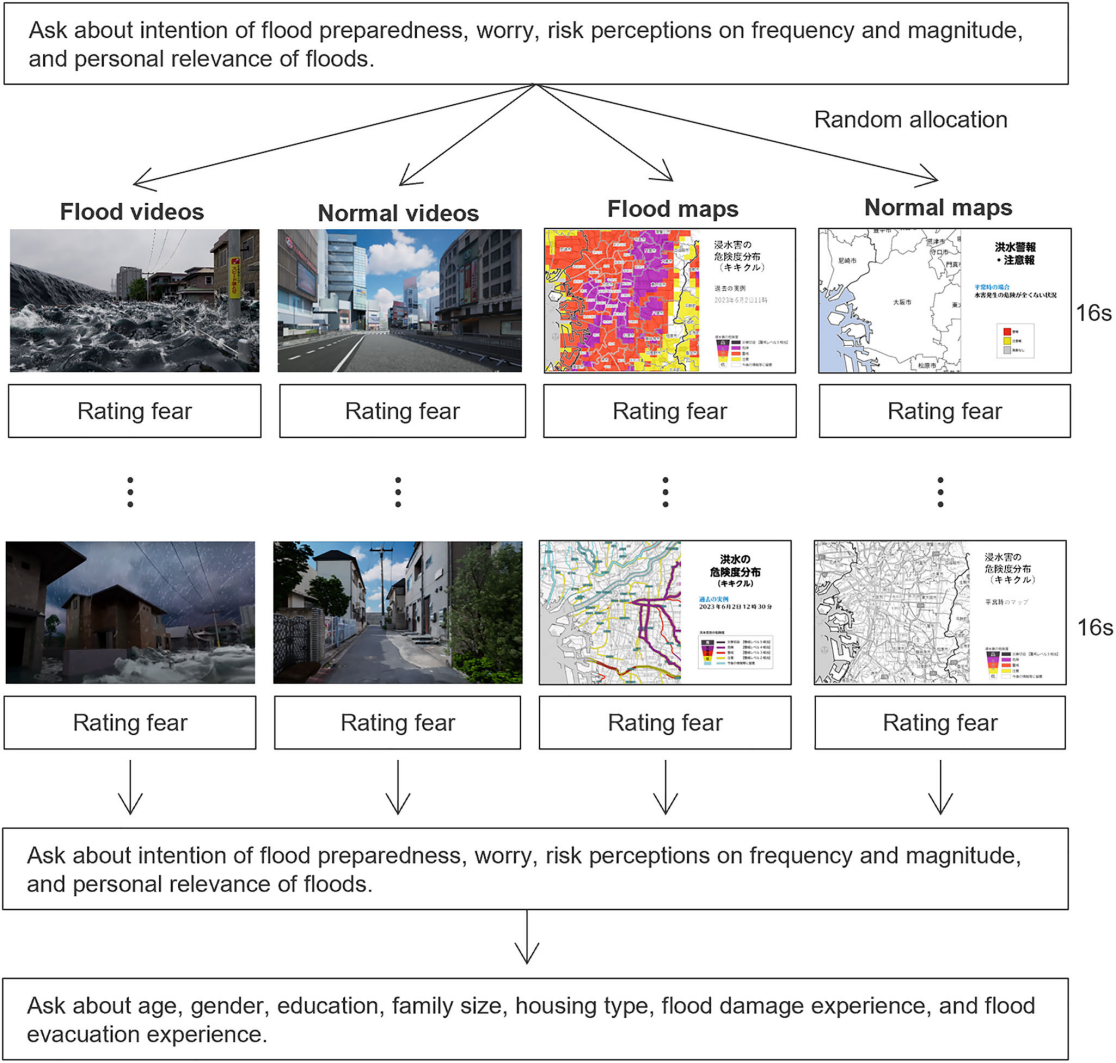
Flow of the web experiment. Participants first reported baseline preparedness intentions and psychological measures. They were then randomly assigned to one of four conditions (flood videos, normal videos, flood maps, normal maps), viewed nine different 16‐s stimuli in random order, and rated fear after each exposure. Post‐stimulus preparedness intentions, psychological measures, and demographics were subsequently collected.

#### 2.1.5 Experimental Measures

We focused on four flood preparedness actions recommended by the Japanese government: (1) making an evacuation plan, (2) stockpiling drinking water, food, and daily necessities, (3) preparing an emergency bag, and (4) purchasing flood insurance (Japan Cabinet Public Affairs Office 2025).^1^ If participants had already implemented any of these actions, we asked about their intention to continue doing so. The intention for each preparedness action was measured on a six‐point scale (1 = Not at all, 6 = Very strongly). For analysis, we used an aggregated measure of “intention of flood preparedness,” calculated as the mean of the four intention scores (Cronbach's *α* = 0.89 before and 0.94 after exposure to the experimental stimuli).

The psychological measures were adapted from constructs and item formulations commonly used in previous studies of hazard risk perception, climate change communication, and flood adaptation. Fear was assessed immediately after each of the nine stimuli using the item “How fearful did you feel?” on a 4‐point scale from 1 = not fearful to 4 = very fearful; the nine ratings were averaged (Dillard et al. 2017). Worry was assessed with the item “How worried are you that your home will be damaged by flooding?” Risk perception was assessed with two items: perceived frequency, “How likely do you think it is that your home will be damaged by flooding in the next 10 years?” and perceived damage severity, “In the event of a flood, how much damage do you think your home would suffer?” Personal relevance was assessed with the item “How relevant do you think flooding is to you?” Worry, risk perception, and personal relevance were rated on 6‐point scales with item‐specific anchors. These measures correspond to affective responses, perceived likelihood and consequences, and risk personalization discussed in previous hazard‐risk research (Trumbo et al. 2016; Wilson et al. 2019; Demuth 2018; Noll et al. 2022). Except for fear, which was repeatedly assessed after each stimulus, these constructs were measured with single‐item indicators rather than multi‐item validated scales. Full details of all measures, including item wording and response scales, are provided in Tables A1, A2, A3.

#### 2.1.6 Statistical Analysis

We conducted preregistered two‐sample *t*‐tests (two‐tailed, α=0.05) to compare changes in flood preparedness intention, worry, risk perceptions (frequency and magnitude), and personal relevance before and after stimulus exposure between the flood video and flood map conditions.

We then conducted a mediation analysis using a regression‐based approach (Baron and Kenny 1986; Hayes 2022) to examine whether flood videos increased flood preparedness intention through fear, worry, risk perceptions, and personal relevance compared with flood maps. Because the web experiment employed a randomized two‐wave pre–post design, it generated repeated observations within individuals. Consistent with our preregistered analytical plan, we specified individual fixed‐effect (FE) panel regression models for the outcome and mediators and estimated these equations simultaneously. This specification follows the standard econometric terminology of FE panel models (Wooldridge 2010). The FE model estimates within‐individual pre–post changes in preparedness intention while removing the influence of unobserved time‐invariant individual heterogeneity such as disaster awareness, risk attitudes, family responsibilities, trust in authorities, and the ability to imagine disaster consequences. Thus, the FE specification provides an additional adjustment for stable individual heterogeneity when estimating changes associated with stimulus exposure.

The preparedness‐intention equation in the simultaneous FE panel regression model is specified as follows:

$$\begin{array}{ccc} y_{it} & = & \beta_{V}\mathrm{Vide}o_{i}\cdot 1_{t}\left[t=2\right]+\beta_{F}\mathrm{Floo}d_{i}\cdot 1_{t}\left[t=2\right]+\beta_{I}\mathrm{Vide}o_{i}\\ & & \times \;\mathrm{Floo}d_{i}\cdot 1_{t}\left[t=2\right]+\gamma \mathrm{Fea}r_{i}\cdot 1_{t}\left[t=2\right]+\delta^{\prime }x_{it}+\mu_{i}+\phi_{t}+\varepsilon_{it}, \end{array}$$

where i denotes a participant and t a time point, with t=1 indicating before and t=2 after stimuli exposure. $y_{it}$ represents the intention of flood preparedness. $\mathrm{Vide}o_{i}$ is a dummy variable (1 if the condition was “videos,” 0 otherwise). $\mathrm{Floo}d_{i}$ is a dummy variable (1 if the condition is “flood situation,” 0 otherwise). $\mathrm{Vide}o_{i}\times \mathrm{Floo}d_{i}$ is the interaction term. $1_{t}\left[t=2\right]$ is a dummy variable (1 if t=2, 0 otherwise). $\mathrm{Fea}r_{i}$ and $x_{it}$ are fear and the other psychological measures, respectively. $\mu_{i}$ and $\phi_{t}$ denote individual and time FEs, respectively. $\varepsilon_{it}$ is an error term. $\beta_{V}$, $\beta_{F}$, $\beta_{I}$, γ, and δ are parameters to be estimated.

Similarly, worry, risk perceptions on frequency and magnitude, and personal relevance were regressed with FE panel models on Flood, Video, Flood × Video, and fear. Specifically, for each psychological measure k, the equation was specified as follows:

$$\begin{array}{ccc} x_{it}^{k} & = & \beta_{V}^{k}\mathrm{Vide}o_{i}\cdot 1_{t}\left[t=2\right]+\beta_{F}^{k}\mathrm{Floo}d_{i}\cdot 1_{t}\left[t=2\right]+\beta_{I}^{k}\mathrm{Vide}o_{i}\\ & & \times \;\mathrm{Floo}d_{i}\cdot 1_{t}\left[t=2\right]+\gamma^{k}\mathrm{Fea}r_{i}\cdot 1_{t}\left[t=2\right]+\mu_{i}^{k}+\phi_{t}^{k}+\varepsilon_{it}^{k} \end{array}$$

where $x_{it}^{k}$ denotes participant i’s score of psychological measure k at time point t. $\mu_{i}^{k}$ and $\phi_{t}^{k}$ denote individual and time FEs, respectively. $\varepsilon_{it}^{k}$ is an error term. $\beta_{V}^{k}$, $\beta_{F}^{k}$, $\beta_{I}^{k}$, and $\gamma^{k}$, are parameters to be estimated.

In addition, fear was regressed on Flood, Video, and Flood × Video using a standard regression model:

$$\;\mathrm{Fea}r_{i}=\beta_{V}^{F}\mathrm{Vide}o_{i}+\beta_{F}^{F}\mathrm{Floo}d_{i}+\beta_{I}^{F}\mathrm{Vide}o_{i}\times \mathrm{Floo}d_{i}+\alpha +\varepsilon_{i}^{F}$$

where α is an intercept and $\varepsilon_{i}^{F}$ is an error term. $\beta_{V}^{F}$, $\beta_{F}^{F}$, and $\beta_{I}^{F}$ are parameters to be estimated. All of these equations were estimated simultaneously with the preparedness‐intention equation.

The model estimation was conducted using the R package lavaan. For implementation in lavaan, the FE specification was transformed into an equivalent system of within‐person change‐score equations for the two‐wave panel data. This transformation removes time‐invariant individual heterogeneity, whereas the time FE is captured by the intercept in the change‐score equations. The resulting simultaneous path model was estimated using robust maximum likelihood estimation. Statistical significance was determined at the two‐tailed 0.05 level.

Finally, the mediation effect of Flood × Video on flood preparedness intention through each psychological variable was estimated using the product method (Baron and Kenny 1986; VanderWeele 2016; Hayes 2022). Because the equations were estimated simultaneously, the direct, indirect, and serial indirect effects were defined and estimated within the same model. For the mediation effects, standard errors, percentile‐based 95% confidence intervals, and *p* values were obtained through 5000 bootstrap resamples using the R package lavaan. Holm‐adjusted *p* values were calculated for the five mediator‐specific total indirect effects through fear, worry, risk perception on frequency, risk perception on magnitude, and personal relevance, whereas Holm correction was not applied to the direct effect.

To assess whether the findings were sensitive to the FE specification, we additionally estimated an ANCOVA‐based simultaneous path model and conducted the corresponding mediation analysis as a robustness check.

### 2.2 Results

We first examined changes in flood preparedness intention scores before and after stimulus exposure across the experimental conditions (Figure 3a). Preregistered two‐sample *t*‐tests indicated that the flood videos significantly increased the intention of flood preparedness compared with the flood maps ($M_{\mathrm{video}}=0.451$, $M_{\mathrm{map}}=0.271$, t(502)=2.40, p=0.017, ${\mathrm{Cohen}}^{\prime }s\;d=0.213$). This effect size indicates a small but statistically significant difference between conditions. We then calculated the mean fear rating for each experimental condition by averaging the ratings across the nine stimuli and compared these mean values between conditions (Figure 3b). Flood videos elicited significantly stronger fear than flood maps ($M_{\mathrm{video}}=3.09$, $M_{\mathrm{map}}=2.22$, t(477)=14.1, p<0.001, ${\mathrm{Cohen}}^{\prime }s\;d=1.26$). No statistically significant differences were observed between the flood videos and flood maps for the other psychological measures (Figure 3c).

**FIGURE 3:**
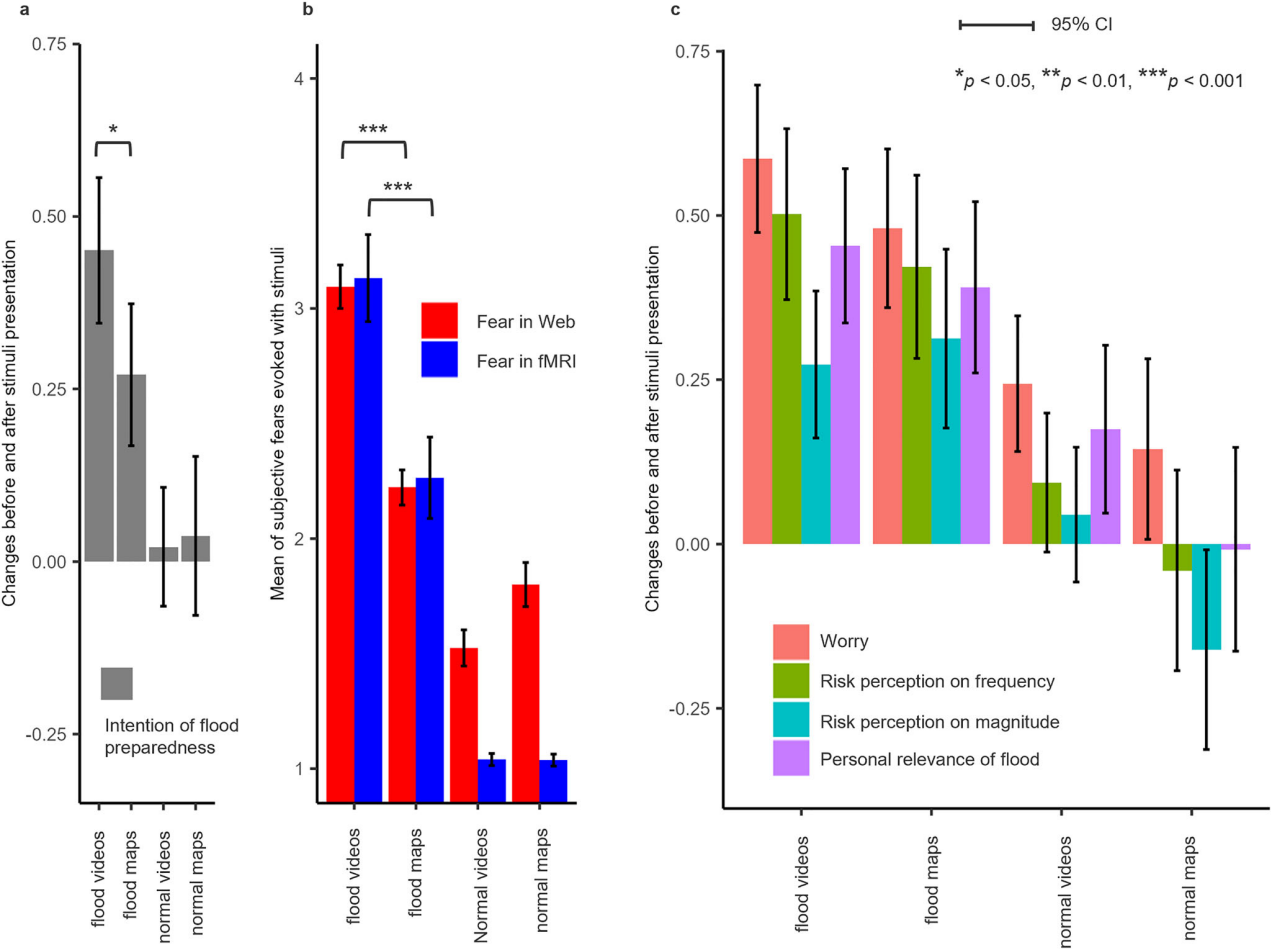
Effects of the four experimental conditions on intention of flood preparedness and fear. (a) Changes in intention of flood preparedness before and after experimental stimuli exposure. (b) Mean fear ratings elicited by the nine stimuli in the web (red) and the fMRI (blue) experiments. (c) Changes in worry, risk perception of flood frequency, risk perception of flood magnitude, and perceived personal relevance of floods before and after stimulus exposure are shown for each condition (flood videos, flood maps, normal videos, normal maps). Error bars indicate 95% confidence intervals.

Mediation analysis was conducted using a regression‐based approach to examine how flood videos increased flood preparedness intention through the psychological measures, compared with flood maps. The estimated path coefficients from the simultaneous FE panel regression model are summarized in Figure 4, with the corresponding full estimation results reported in Table S1 available on OSF: https://osf.io/2sw8a. As shown in Figure 4, the Flood × Video interaction significantly increased fear (b=1.149, p<0.001), whereas it had no significant direct effect on the within‐individual change in preparedness intention and showed negative effects on the within‐individual changes in the other psychological measures.

**FIGURE 4:**
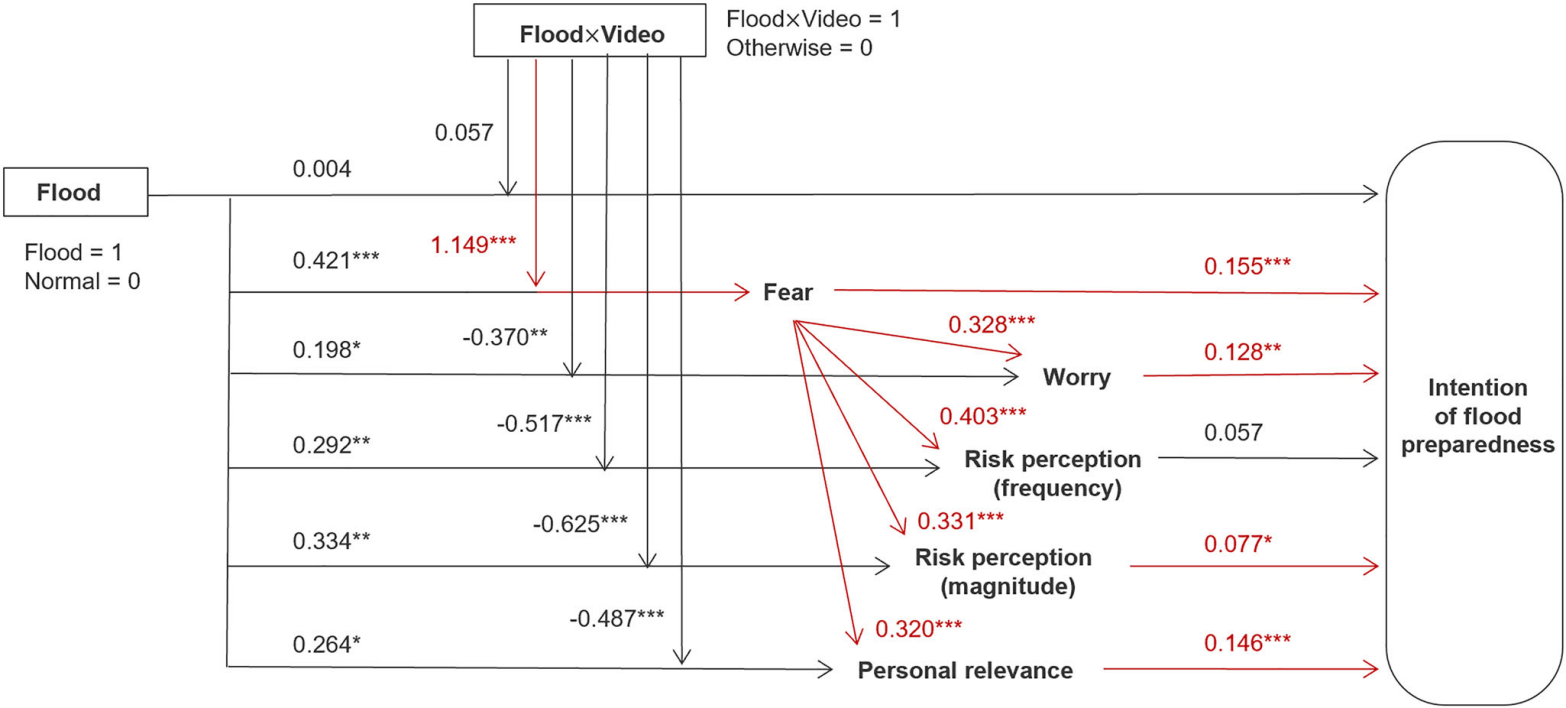
Unified fixed‐effects path model of psychological pathways linking Flood × Video to flood preparedness intention. The model simultaneously estimated the direct and indirect effects of Flood × Video on changes in flood preparedness intention through fear, worry, risk perceptions, and personal relevance. Values are unstandardized path coefficients estimated with robust maximum likelihood standard errors. Red arrows highlight significant path coefficients involved in fear‐related pathways. Paths from Video and residual correlations among mediators are omitted for clarity. *p < 0.05, **p < 0.01, ***p < 0.001.

In the simultaneously estimated FE path model, fear significantly increased the intention of flood preparedness (b=0.155, p<0.001). Combined with the positive path from Flood × Video to fear, this finding suggests that flood videos increased the intention of flood preparedness through fear, compared with flood maps. Fear also had significant positive effects on the other psychological measures: worry (b=0.328, p<0.001), risk perception on frequency (b=0.403, p<0.001), risk perception on magnitude (b=0.331, p<0.001), and personal relevance (b=0.320, p<0.001). Furthermore, the intention of flood preparedness increased with changes in worry (b=0.128, p<0.01), risk perception on magnitude (b=0.077, p<0.05), and personal relevance (b=0.146, p<0.001).

We estimated the mediator‐specific total indirect effects of Flood × Video through fear, worry, risk perceptions (on frequency and magnitude), and personal relevance on the intention of flood preparedness using the product method (Baron and Kenny 1986; VanderWeele 2016; Hayes 2022). Holm correction was applied to the five mediator‐specific total indirect effects. As shown in Table 1, the total indirect effect through fear was statistically significant (b=0.335, 95% CI [0.231, 0.446], Holm‐adjusted p<0.001), indicating that the effect of flood videos on preparedness intention was mediated through fear, compared with flood maps. In contrast, the total indirect effects through worry, risk perceptions, and personal relevance were not statistically significant after Holm correction. In addition, the direct effect of Flood × Video on preparedness intention was not statistically significant after accounting for the measured psychological variables. Thus, among the measured mediators, only the total indirect effect through fear remained statistically significant after Holm correction.

**TABLE 1:** Direct and mediator‐specific total indirect effects of the Flood × Video interaction on preparedness intention.

Mediation pathways	Estimate	SE	95% CI	*p* value	Holm‐adjusted *p*
Flood × Video → (direct) → Intention	0.057	0.102	[−0.143, 0.257]	0.577	—
Flood × Video → Fear → Intention	0.335	0.055	[0.231, 0.446]	<0.001	<0.001
Flood × Video → Worry → Intention	0.001	0.017	[−0.032, 0.038]	0.960	1.000
Flood × Video → Risk perception (frequency) → Intention	−0.003	0.009	[−0.025, 0.014]	0.745	1.000
Flood × Video → Risk perception (magnitude) → Intention	−0.019	0.014	[−0.051, 0.002]	0.168	0.673
Flood × Video → Personal relevance → Intention	−0.017	0.020	[−0.056, 0.023]	0.379	1.000

*Note*: Estimates represent mediator‐specific total indirect effects. For worry, risk perception, and personal relevance, the estimates include indirect pathways in which fear is an upstream mediator. These effects are therefore not mutually exclusive and should not be summed. Holm‐adjusted *p* values were calculated across the five mediator‐specific total indirect effects, excluding the direct effect.

As a robustness check, the ANCOVA‐based simultaneous path model yielded results highly consistent with those of the simultaneous FE model; the full results are provided in Tables S4 and S5 available on OSF: https://osf.io/2sw8a

### 2.3 Discussion

We investigated whether first‐person 3DCG flood videos increased flood preparedness intention compared with flood maps and which psychological factors mediated this effect, focusing on fear, worry, risk perceptions, and personal relevance. The results indicated that flood videos produced a small but statistically significant increase in preparedness intention compared with flood maps. The simultaneous FE mediation analysis further showed that this effect was primarily explained by fear evoked by the experimental stimuli. Specifically, after applying Holm correction to the five mediator‐specific total indirect effects, only the indirect effect through fear remained statistically significant; the indirect effects through worry, risk perception on frequency, risk perception on magnitude, and personal relevance were not statistically significant. The direct effect of Flood × Video on preparedness intention was also not statistically significant after accounting for the measured psychological variables. Thus, the mediation results support a specific fear‐mediated pathway rather than a broader mediation process involving all measured psychological variables.

The finding that flood videos strongly affected fear but did not produce corresponding increases in worry, risk perceptions, or personal relevance may reflect differences in construct dynamics. Fear was measured as an immediate emotional reaction to each stimulus, whereas the other variables reflected participants’ evaluations of their own flood risk after exposure. Thus, flood videos may have primarily influenced stimulus‐evoked fear, whereas changes in broader self‐relevant risk appraisals may require additional reflection, stronger personal connection, or repeated exposure.

## 3 fMRI Experiment

The study hypothesis was then examined at the neural level. Because fear played a central role in the web experiment results, we predicted that flood videos would elicit greater activation in brain regions associated with fear compared with flood maps. Fear‐related brain regions are well established, and meta‐analyses of neuroimaging studies consistently identify the amygdala (Phan et al. 2002; Murphy et al. 2003; Vytal and Hamann 2010; Lindquist et al. 2012; Ipser et al. 2013; García‐García et al. 2016; Gentili et al. 2019; Tao et al. 2021), insula (Murphy et al. 2003; Vytal and Hamann 2010; Lindquist et al. 2012; Ipser et al. 2013; Gentili et al. 2019), anterior cingulate cortex (ACC) (Murphy et al. 2003; Vytal and Hamann 2010; Lindquist et al. 2012), and ventromedial prefrontal cortex (vmPFC) (Phan et al. 2002; Murphy et al. 2003; Lindquist et al. 2012) as key areas. Several studies have further shown that activity in these regions is associated with subjective fear experiences (Stark et al. 2007; Zhou et al. 2021). Accordingly, we investigated whether the flood videos would show greater activation in the amygdala, ACC, vmPFC, and insula—defined as regions of interest (ROIs)—than flood maps.

### 3.1 Method

We conducted an fMRI experiment with 45 participants residing in Osaka City—the same city as in the web experiment—between November 18 and 29, 2024. The study protocol was approved by the Ethical Committee of Kyoto University Medical School (protocol #R4603). The preregistration of this study is available on the OSF (https://osf.io/2sw8a).

#### 3.1.1 Participants

The experiment involved 45 healthy, right‐handed participants (age: M=41.2, SD=11.8; 22 women, 23 men). Their demographic characteristics were similar to those of the web experiment sample (Table A2). All participants provided written informed consent for the procedure. Participants were recruited through Chuo‐Sato Inc., a company with extensive experience in supporting fMRI studies. Individuals with a history of drug use, psychiatric disorders, or life‐threatening flood experiences were excluded. Each participant received financial compensation of 12,000 yen (approximately $80) after completing the experiment. Because no prior fMRI studies on flood risk were available, the sample size was heuristically set at 45, based on reference to previous studies on naturalistic risks (Megías et al. 2018; Oba et al. 2022) and considering financial resource constraints (Lakens 2022).

#### 3.1.2 Design and Stimuli

The fMRI experiment employed a within‐subjects 2 × 2 factorial block design. As in the web experiment, the first factor was the type of medium (videos vs. maps), and the second factor was the type of situation (flood vs. normal). All stimuli used in the fMRI experiment were identical to those in the web experiment.

#### 3.1.3 Experimental Procedure

The fMRI experiment comprised six sessions, each consisting of 12 blocks, for a total of 72 blocks. The procedure for each session is illustrated in Figure 5. Each session began with a 10‐s fixation cross, followed by 12 blocks presented in succession. One of the four experimental conditions (flood and normal videos, and flood and normal maps) was pseudo‐randomly assigned to each block; each stimulus within the assigned condition was also pseudo‐randomly selected for presentation to the participant. Thus, each stimulus was presented twice to achieve counterbalance over the 72 blocks in the experiment. In each block, a participant was presented with the stimulus for 16 s on the screen equipped with an MRI machine. Then, during the subsequent 4 s, the participant was required to answer the question “How fearful did you feel?” displayed on the screen with four‐point scales (1: not fearful–4: very fearful) by using a response pad in their right hand. The block ended with a fixation cross for 12 s. Stimuli presentation was controlled using Presentation Software (version 24.0). The stimuli were projected from a liquid crystal projector to a mirror positioned in a scanner in front of the participant. After the fMRI experiment, we asked about participants’ demographics (age, gender, education, family size, and housing type), and flood evacuation experience and damage experiences.

**FIGURE 5:**
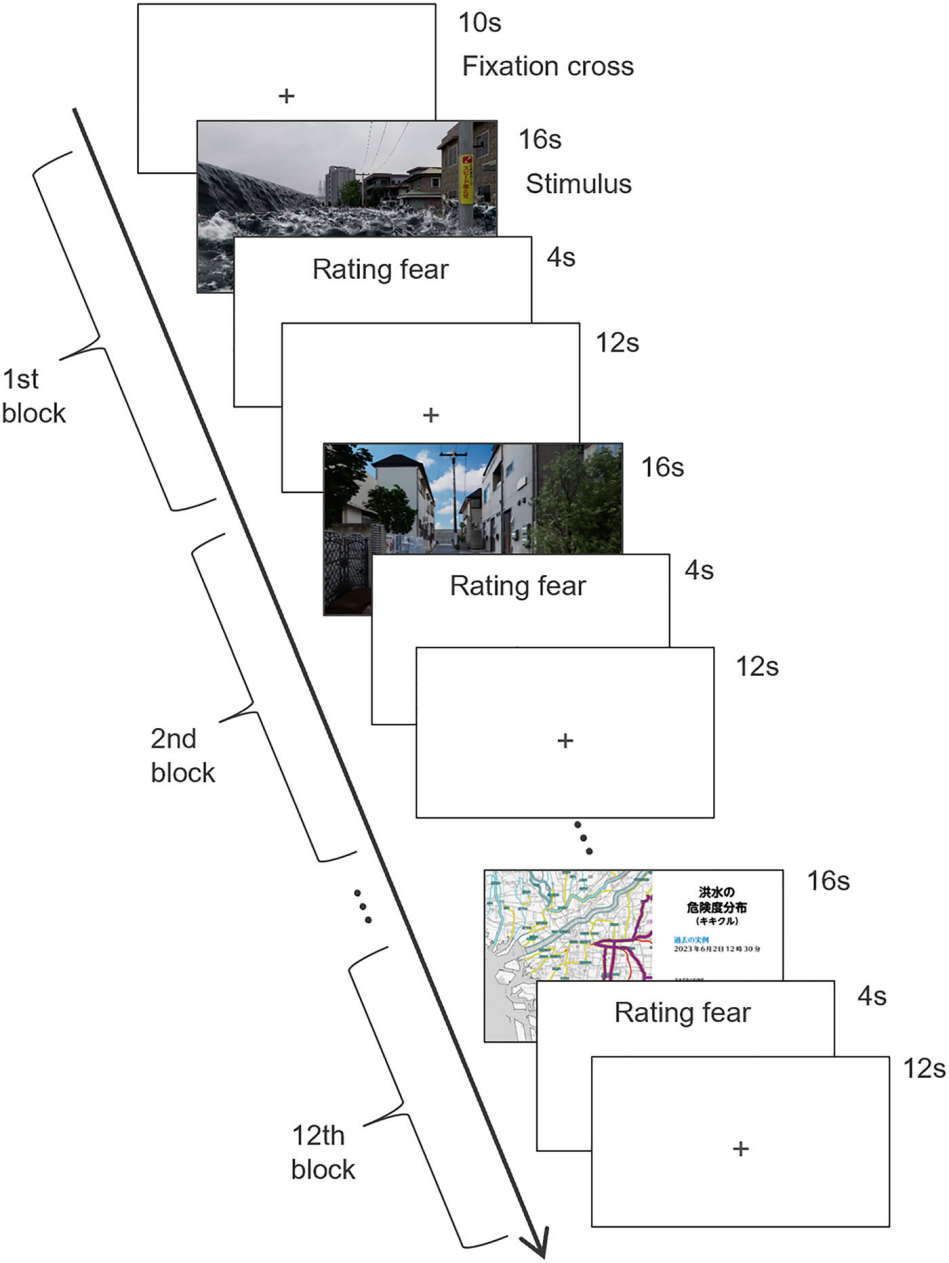
fMRI experiment process flow in each session. The fMRI experiment consisted of six sessions. Each session included 12 blocks. In each block, a participant was presented with a pseudo‐randomly chosen stimulus and asked to rate subjective fear for it.

#### 3.1.4 Data Acquisition

The fMRI data were acquired at the ATR‐Promotions Brain Activity Imaging Center using a 3‐T scanning system (MAGNETOM Prisma) with a 64‐channel head coil. Small elastic pads were placed on both sides of the head to stabilize its position. The functional images consisted of 66 consecutive axial slices parallel to the anterior commissure–posterior commissure plane and covering the whole brain. A T2*‐weighted multiband gradient‐echo echo‐planar imaging (EPI) sequence was used with the following parameters: repetition time (TR) = 2000 ms; echo time (TE) = 30 ms; flip angle = 80°; multiband acceleration factor = 3; partial Fourier = 6/8; matrix size = 100 × 100; voxel size = 2 × 2 × 2 mm. At the beginning of each fMRI session, we acquired a gradient‐echo field map to correct for geometric distortions (TR = 750 ms; TE1/TE2 = 5.17/7.63 ms [ΔTE = 2.46 ms]; flip angle = 50°; matrix size = 100 × 100; 66 slices in the same orientation and geometry as the EPI sequence). After acquiring the functional images, a T1‐weighted high‐resolution anatomical image was obtained using a magnetization prepared rapid‐acquisition gradient‐echo (MPRAGE) sequence (TR = 2250 ms; TE = 3.06 ms; inversion time = 900 ms; flip angle = 9°; GRAPPA acceleration factor = 2; 208 sagittal slices; slice thickness = 1 mm; field of view = 256 × 256 mm; voxel size = 1 × 1 × 1 mm).

#### 3.1.5 Data Pre‐Processing

All preprocessing and statistical analysis of fMRI data were performed using SPM12 implemented in MATLAB. For preprocessing, all functional images were first corrected for slice timing. Next, all functional images were motion‐corrected via spatial realignment to the first volume and were unwarped to correct for distortions based on the field map using FieldMap Toolbox (Andersson et al. 2001). The anatomical images were then coregistered to the mean functional image. These images were spatially normalized to the standard Montreal Neurological Institute (MNI) space using the anatomical image‐based unified segmentation‐spatial normalization approach (Ashburner and Friston 2005) and resampled to a resolution of 2 × 2 × 2 mm. Finally, the functional images were spatially smoothed with a 6 mm full width at half‐maximum (FWHM) isotropic Gaussian kernel to compensate for anatomical variability among participants.

#### 3.1.6 Data Analysis

A two‐step random effect general linear model was used for the statistical analysis of the fMRI data (Holmes and Friston 1998). In the first‐level single‐subject model, four experimental conditions (flood and normal videos, and flood and normal maps) were embedded within a series of boxcar functions convolved with the canonical hemodynamic response function. The rating task after the presentation of experimental conditions was also modeled as the effect of no interest. A high‐pass filter with a cutoff period of 128 s was used to eliminate the artifactual low‐frequency noise. Six motion parameters estimated from spatial realignment in the preprocessing steps and nuisance regressors from the white matter and cerebrospinal fluid based on the CompCor method (Behzadi et al. 2007) were added to the model to reduce motion‐related artifacts and physiological noise using the PhysIO Toolbox (Kasper et al. 2017). Serial autocorrelation was corrected using an AR(1) model. The contrast images of [flood videos—normal videos] and [flood maps—normal maps] from each participant's first‐level analysis were entered into a full factorial model for the second‐level random effects (group) analysis. Corrections for non‐sphericity were applied to account for the potential non‐sphericity of repeated measures data using the variance component model. We conducted an ROI analysis based on our prior anatomical hypothesis using small volume correction (SVC) (Worsley et al. 1996) for the contrast of [flood videos—normal videos] > [flood maps—normal maps], inclusively masked by the contrast of [flood videos—normal videos] (at p<0.05 uncorrected) to ensure that only interaction with respect to the positive effect of the video conditions was evaluated. Here, the ROIs were set to be bilateral amygdala, insula, ACC, and vmPFC, defined using the AAL3 atlas (Tzourio‐Mazoyer et al. 2002; Rolls et al. 2020). The statistical threshold was set to p<0.05 with a peak‐level familywise error (FWE) corrected for multiple comparisons over each ROI.

In addition, we conducted a supplementary parametric modulation analysis to assess the relationships between brain activity and subjective fear ratings. For this analysis, four experimental conditions were modeled as stimulus presentation events, and fear ratings were incorporated as parametric modulators of these events. For the first‐level single‐subject model, we maintained the same preprocessing and noise‐reduction settings as the main analysis, including high‐pass filtering, serial autocorrelation correction, and nuisance regression. We then subjected the individual stimulus presentation and parametric modulation contrast images to a standard second‐level group analysis. The same ROIs and statistical threshold were used with the main analysis.

### 3.2 Results

The subjective fear ratings for flood videos and maps are shown in Figure 3b. The Flood × Video interaction on subjective fear was significant (F(1176)=42.2, p<0.001, partial $\eta^{2}=0.193$), consistent with the web experiment. Planned contrasts further confirmed that flood videos elicited significantly stronger subjective fear than flood maps ($M_{\mathrm{video}}=3.12$, $M_{\mathrm{map}}=2.26$, t(88)=6.58, p<0.001, ${\mathrm{Cohen}}^{\prime }s\;d=1.39$).

The results of regional brain activity for the condition effects revealed a significant Flood × Video interaction in the right amygdala (x=26, y=2, z=−26; T(88)=3.73; p<0.05 SVC family‐wise error‐corrected), as illustrated in Figure 6. The bar graph indicates that the amygdala was activated in response to the flood versus normal videos, but not to the flood versus normal maps. No significant activations were observed in the other ROIs. The parametric modulation analysis of subjective fear ratings showed modest activation in the right amygdala that did not reach significance (x=26, y=2, z=−28; T(88)=2.43; p<0.01, uncorrected peak‐level threshold), showing a trend of association between amygdala activity and subjective fear.

**FIGURE 6:**
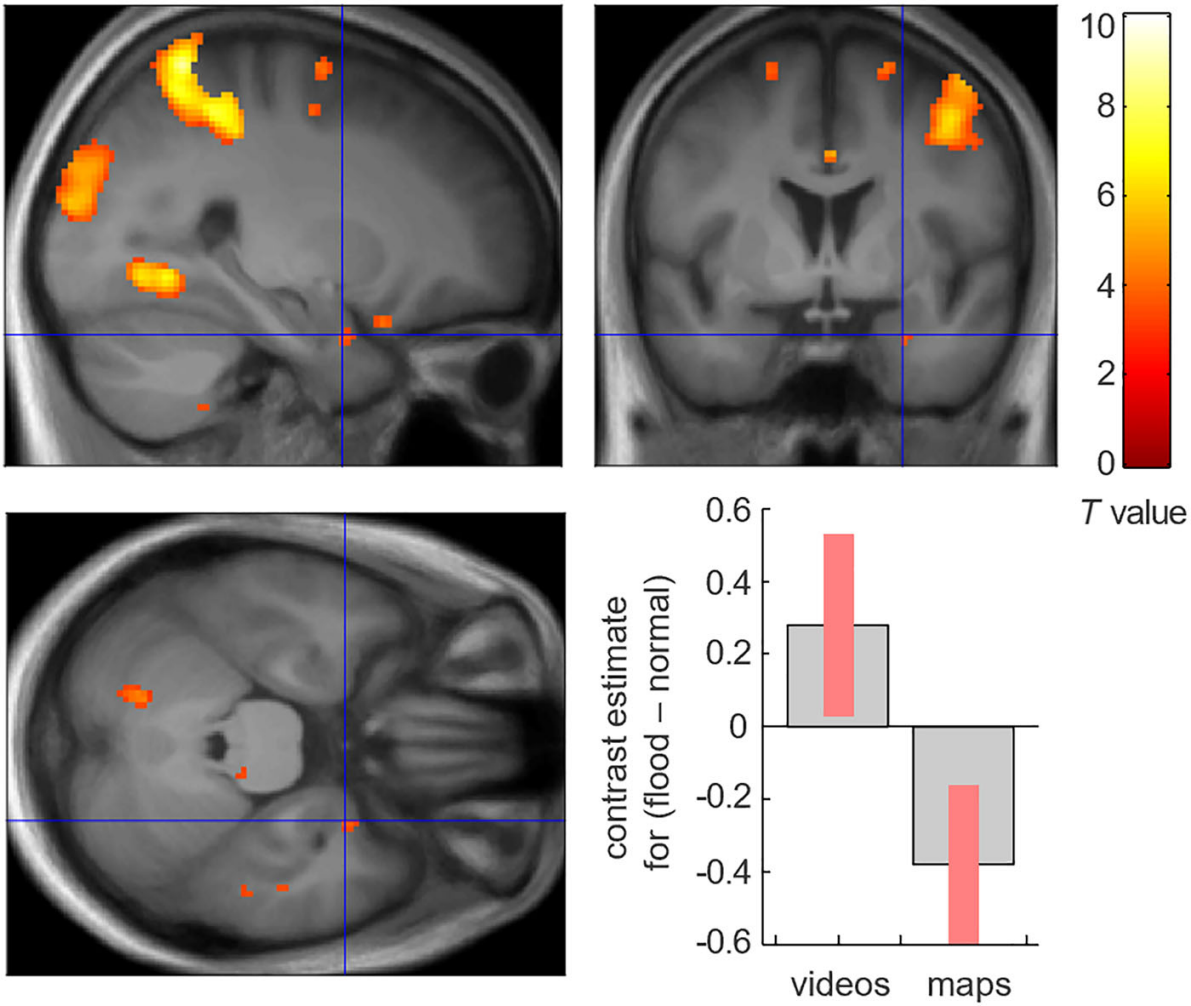
Brain imaging results. Group statistical parametric maps. Threshold is *p* < 0.001 for display purposes, and mean (±90% confidence interval) effect size indicates the significant interaction between video and flood. The blue cross indicates the location of the peak voxel in the right amygdala (x=26, y=2, z=−26; T(88)=3.73; p<0.05 SVC family‐wise error‐corrected). The area is superposed on mean normalized structural images of the participants in this study.

### 3.3 Discussion

The fMRI experiment examined differences in neural responses to first‐person 3DCG flood videos and flood maps, with a focus on fear‐related ROIs. The ROI analysis revealed a significant Flood × Video interaction in the right amygdala: right amygdala activity was greater for flood versus normal videos, whereas no corresponding difference was observed for flood versus normal maps. No significant activations were observed in the other predefined ROIs. We speculate that only the amygdala was active among our ROIs of the fear‐related brain regions because our flood video particularly stimulated the Type 1 processing. The amygdala is a subcortical structure involved in survival circuits and is widely associated with emotional salience and fear‐related processing (Adolphs 2013; LeDoux and Pine 2016; Fanselow 2018). Some neuroimaging studies have found that visual emotional stimuli were processed automatically through the subcortical visual pathway to the amygdala, which includes the superior colliculus and pulvinar (Morris et al. 1999; Pasley et al. 2004; Williams et al. 2006; Sato et al. 2019). Other studies showed that the amygdala was activated in response to emotional stimuli independently of conscious evaluation of stimuli (Pierce et al. 2022; Bo et al. 2024). Meta‐analyses of neuroimaging studies reported reliable amygdala activation in response to fear‐related stimuli (e.g., García‐García et al. 2016; Gentili et al. 2019; Tao et al. 2021). Although the parametric modulation analysis of subjective fear ratings showed only a modest, non‐significant trend in the right amygdala, the ROI result is consistent with the interpretation that first‐person 3DCG flood videos elicited stronger fear‐related responses than flood maps.

## 4 General Discussion

This study examined how first‐person 3DCG flood videos, as one operationalization of experiential flood‐risk communication, affect individual flood preparedness intentions compared with flood maps, as a common form of descriptive flood‐risk communication. By combining large‐scale web‐based behavioral data with functional neuroimaging, we provide convergent behavioral and neural evidence that first‐person 3DCG flood videos evoke stronger fear‐related responses than flood maps.

The web experiment indicated that subjective fear helped explain a small but statistically significant effect of flood videos on preparedness intentions. This result is consistent with prior work suggesting that affective responses to vivid risk information can contribute to preparedness intentions (Loewenstein et al. 2001; Leiserowitz 2006; Weber 2006). Fear was positively associated with worry, risk perceptions, and personal relevance, suggesting that stimulus‐evoked fear may be related to broader self‐relevant risk appraisals. However, the statistically supported indirect pathway from Flood × Video to preparedness intention was specific to fear.

The fMRI experiment provided corroborative evidence that flood videos elicited greater activation in the right amygdala, a region associated with fear‐related processing. Considering that the amygdala is known to detect emotional salience and trigger emotional responses (Adolphs 2013; Fanselow 2018), these findings are consistent with the interpretation that first‐person 3DCG flood videos elicited stronger fear‐related responses than flood maps. Together with the web experiment, the fMRI result provides convergent evidence supporting a fear‐based interpretation of the observed increase in preparedness intentions.

These findings can be interpreted within the framework of dual‐process theories of risk judgment (Stanovich and West 2000; Evans and Stanovich 2013). Flood maps, as descriptive information, convey risk through symbolic and spatial representations that require residents to interpret the information and translate it into personally relevant consequences (Fujimi et al. 2026). In contrast, first‐person 3DCG flood videos, as experiential information, may make flood risk perceptually concrete and emotionally salient, thereby more strongly engaging rapid, affective, and associative Type 1 processing (Fujimi et al. 2026). Such affective engagement may have provided an initial pathway through which the videos contributed to preparedness intentions.

The study findings have implications for flood‐risk communication in climate adaptation strategies. Current communication practices often rely on descriptive information, such as flood maps, which may be difficult for some residents to translate into concrete images of flooding. Our findings suggest that first‐person 3DCG flood videos may complement map‐based communication by increasing emotional salience and helping residents imagine flooding more vividly. At the same time, effective flood‐risk communication should not rely solely on Type 1 affective engagement; Type 2 deliberative processing remains important for interpreting hazard information, evaluating feasible preparedness options, and translating emotional salience into concrete preparedness plans. Such integration could foster more inclusive, accessible, and actionable preparedness strategies, particularly in urban areas facing increasing flood risks due to climate change.

Moreover, this study contributes to the growing discourse on the ethical design of risk communication. Although fear played a central role in the observed effect, experiential flood‐risk information differs meaningfully from fear appeal messages. Fear appeals typically use threat and fear arousal as part of a persuasive strategy to motivate protective action, often by emphasizing threat severity, susceptibility, and recommended responses (Witte and Allen 2000; Tannenbaum et al. 2015). In contrast, the experiential information examined in this study was designed to provide accurate and perceptually vivid representations of possible flooding situations based on official flood‐risk information, rather than to rely on fear arousal as an explicit persuasive strategy. This distinction is ethically important because fear‐based communication can raise concerns about adverse reactions, such as message avoidance, denial of the threat, or resistance to recommended actions (Hastings et al. 2004; Ruiter et al. 2014). Prior research has shown that participants often perceive virtual flood experiences positively, even when they evoke negative emotions (Simpson et al. 2022; D'Amico et al. 2023; Wilhelmi et al. 2024), suggesting that experiential information may offer a promising and potentially responsible way to engage the public in risk adaptation.

Although this study focused on flood preparedness, the theoretical and methodological approach may be extended to other climate‐related hazards, such as wildfires, heatwaves, or sea‐level rise. By elucidating the neurobehavioral mechanisms through which different types of risk information shape individual responses, this research supports the development of more effective, science‐informed policies for disaster preparedness and long‐term climate resilience. Future work should explore how experiential information can be incorporated into public education campaigns, participatory planning tools, and digital governance platforms to empower communities in adapting to accelerating climate threats.

Several limitations should be acknowledged. First, we examined first‐person 3DCG flood videos only in the context of urban flood risk, and replication in other hazards is needed to test generalizability. Second, our outcomes focused on preparedness intentions rather than actual behaviors; future work should examine whether the emotional and neural responses observed here are associated with real‐world preparedness actions. Third, experiential information was operationalized as first‐person 3DCG flood videos, which combined multiple characteristics, including dynamic presentation, first‐person perspective, realism, and vividness. Consequently, the present study cannot disentangle the relative contribution of these characteristics to the observed behavioral and neural effects. Relatedly, all stimuli were presented without sound. Although this choice was made to maintain comparability between the video and map conditions, the absence of auditory cues may have influenced emotional responses, especially for the video condition, by altering the naturalness of the scenes or increasing uncertainty and perceived threat. Future studies should isolate and quantify the relative contribution of features such as motion, perspective, realism, vividness, and auditory cues to emotional engagement and preparedness. Fourth, although we conceptually distinguished experiential information from fear appeals, this distinction was not empirically tested; direct comparisons could clarify not only emotional and neural responses but also possible adverse effects such as message avoidance. Addressing these limitations would further solidify the empirical foundation for neuroscience‐informed risk communication.

In summary, this study provides convergent behavioral and neural evidence that first‐person 3DCG flood videos elicit stronger fear‐related responses than flood maps. The web experiment showed that flood videos produced a small but statistically significant increase in preparedness intentions, with subjective fear helping explain this increase. The fMRI experiment further showed greater right amygdala activation for flood videos than flood maps, consistent with this fear‐based interpretation. These findings suggest that visually vivid flood videos may complement conventional map‐based communication by increasing emotional salience and promoting flood‐preparedness intentions.

## Conflicts of Interest

The authors declare no conflicts of interest.

## Supporting information




**Supplementary information**: risa70407‐supp‐0001‐SuppMat.pdf

## Data Availability

Preregistration and all stimuli are available at OSF (https://osf.io/a4xbm). Data and analysis code are openly accessible at https://osf.io/rcfyd/files.

## Endnotes

1 In the preregistration, we originally planned to measure “intention of flood preparedness” as the average of six preparedness actions, including two additional actions: (5) checking a flood risk map of the residential area and (6) checking flood warning maps in the event of flood danger. However, these two actions were excluded from the current analysis because they overlapped with the experimental stimuli of the flood maps, which could have compromised fair comparisons across conditions. To assess the impact of this modification, we conducted an additional analysis using the six‐action version of “intention of flood preparedness.” The results were highly consistent with those obtained from the four‐action version used in this study, as reported in Figure S1 and Tables S2 and S3 available on OSF: https://osf.io/2sw8a

## 1

**TABLE A1:** Definitions of variables used in the web and fMRI experiment.

Variable	Definition
**Demographic variables**	
Age	Year
Female	Female = 1, male = 0
Family size	The number of family members living together
University	Graduation of university or graduate school = 1, otherwise = 0
House	Living in a detached house = 1, otherwise = 0
Flood damage experience	Having experience of flood damage = 1, otherwise = 0
Flood evacuation experience	Having experience of flood evacuation = 1, otherwise = 0
Evacuation plan	Answers to “Do you have an evacuation plan for you or your family in case of flooding?” Yes = 1, No = 0
Emergency stocks	Answers to “Do you have a stockpile of drinking water, food, and daily necessities in case of flooding?” Yes = 1, No = 0
Emergency bag	Answers to “Do you have an emergency bag in case of flooding?” Yes = 1, No = 0
Insurance	Answers to “Do you have flood insurance?” Yes = 1, No = 0
**Psychological variables**	
Intention of flood preparedness	The average score of intentions of the following four items: evacuation plan, emergency stocks, emergency bag, and insurance
Intention: evacuation plan	Answers to “How willing would you be to make an evacuation plan for you or your family in case of flooding? If you already have an evacuation plan, please indicate how willing you would be to keep it.” [6‐point scale] (1) Not at all—(6) Very strongly
Intention: emergency stocks	Answers to “How much would you be willing to stockpile drinking water, food, and daily necessities in preparation for flooding? If you are already stockpiling, please indicate how much you would be willing to continue doing so.” [6‐point scale] (1) Not at all—(6) Very strongly
Intention: emergency bag	Answers to “How willing would you be to prepare an emergency bag in case of flooding? If you have already prepared one, please indicate how willing you would be to continue doing so.” [6‐point scale] (1) Not at all—(6) Very strongly
Intention: insurance	Answers to “How willing would you be to purchase flood insurance? If you already have flood insurance, please indicate how willing you would be to continue to keep it.” [6‐point scale] (1) Not at all—(6) Very strongly
Fear	The average of nine scores of fear ratings for the nine stimuli. Each score was rated by answering “How fearful did you feel?” just after presentation of each stimulus. [4‐point scale] (1) Not fearful—(4) Very fearful
Worry	Answers to “How worried are you that your home will be damaged by flooding?” [6‐point scale] (1) Not at all—(6) Very strongly
Risk perception on frequency	Answers to “How likely do you think it is that your home will be damaged by flooding in the next 10 years?” [6‐point scale] (1) Not at all—(6) Very likely
Risk perception on magnitude	Answers to “In the event of a flood, how much damage do you think your home would suffer?”[6‐point scale] (1) Not at all—(6) Very large
Personal relevance	Answers to “How relevant do you think flooding is to you?” [6‐point scale] (1) Not at all—(6) Very strongly

**TABLE A2:** Descriptive statistics and balance across experimental conditions.

Variable	Web experiment									fMRI (45)	
	Flood video (249)		Flood map (256)		Normal video (246)		Normal map (249)		Balance test		
	Mean	SD	Mean	SD	Mean	SD	Mean	SD		Mean	SD
Age	43.7	10.5	44.0	10.3	43.5	10.2	42.7	10.2	*F* = 0.734	41.2	11.8
Female	0.53	0.50	0.60	0.49	0.50	0.50	0.57	0.50	*F* = 1.893	0.49	0.51
Family size	2.27	1.33	2.38	1.25	2.26	1.26	2.26	1.25	*F* = 0.545	2.38	1.15
University	0.45	0.50	0.46	0.50	0.46	0.50	0.51	0.50	*F* = 0.590	0.71	0.46
House	0.29	0.45	0.26	0.44	0.24	0.43	0.27	0.45	*F* = 0.564	0.24	0.44
Flood damage experience	0.04	0.20	0.04	0.19	0.05	0.21	0.04	0.20	*F* = 0.099	0.07	0.25
Flood evacuation experience	0.04	0.19	0.04	0.20	0.02	0.16	0.04	0.19	*F* = 0.439	0.02	0.15
Evacuation plan	0.24	0.43	0.23	0.42	0.22	0.42	0.27	0.44	*F* = 0.449	0.29	0.46
Emergency stocks	0.45	0.50	0.48	0.50	0.46	0.50	0.49	0.50	*F* = 0.200	0.49	0.51
Emergency bag	0.33	0.47	0.31	0.46	0.32	0.47	0.38	0.49	*F* = 1.059	0.36	0.48
Insurance	0.17	0.38	0.15	0.36	0.13	0.34	0.17	0.38	*F* = 0.805	0.11	0.32

*Note*: The number in parentheses is the sample size. “SD” indicates standard deviation. The “Balance” column shows analysis of variance (ANOVA) *F* values.

^*^ *p* < 0.05.

^**^ *p* < 0.01.

^***^ *p* < 0.001.

**TABLE A3:** Psychological measures before and after stimulus exposure in the web experiment.

	Flood video (249)		Flood map (256)		Normal video (246)		Normal map (249)		Balance
Variable	Mean	SD	Mean	SD	Mean	SD	Mean	SD	Test
**Before stimuli presentation**									
Intention of flood preparedness	3.20	1.20	3.19	1.06	3.24	1.16	3.26	1.12	*F* = 0.179
Intention: evacuation plan	3.23	1.33	3.09	1.17	3.17	1.33	3.22	1.23	*F* = 0.614
Intention: emergency stocks	3.41	1.38	3.47	1.23	3.50	1.36	3.47	1.30	*F* = 0.209
Intention: emergency bag	3.35	1.41	3.39	1.24	3.42	1.37	3.50	1.34	*F* = 0.596
Intention: insurance	2.82	1.30	2.80	1.31	2.85	1.32	2.83	1.34	*F* = 0.061
Worry	2.77	1.26	2.82	1.19	2.65	1.22	2.79	1.36	*F* = 0.816
Risk perception on frequency	3.00	1.24	3.14	1.20	2.97	1.23	3.07	1.30	*F* = 0.921
Risk perception on magnitude	3.32	1.35	3.32	1.24	3.24	1.26	3.39	1.36	*F* = 0.577
Personal relevance	3.02	1.39	3.03	1.28	2.84	1.32	3.12	1.41	*F* = 1.795
**After stimuli presentation**									
Intention of flood preparedness	3.65	1.29	3.46	1.18	3.26	1.25	3.29	1.26	*F* = 5.252***
Intention: evacuation plan	3.66	1.39	3.44	1.25	3.24	1.34	3.29	1.36	*F* = 4.807***
Intention: emergency stocks	3.76	1.38	3.68	1.26	3.42	1.37	3.39	1.37	*F* = 4.777***
Intention: emergency bag	3.73	1.40	3.53	1.33	3.36	1.38	3.39	1.40	*F* = 3.700**
Intention: insurance	3.46	1.45	3.19	1.44	3.01	1.38	3.10	1.41	*F* = 4.728***
Fear	3.09	0.76	2.22	0.62	1.52	0.63	1.80	0.77	*F* = 239.2^***^
Worry	3.36	1.33	3.30	1.23	2.90	1.20	2.93	1.39	*F* = 8.726***
Risk perception on frequency	3.50	1.23	3.56	1.16	3.06	1.16	3.03	1.34	*F* = 13.287***
Risk perception on magnitude	3.59	1.29	3.63	1.12	3.28	1.23	3.23	1.34	*F* = 6.950***
Personal relevance	3.47	1.34	3.42	1.32	3.02	1.29	3.11	1.42	*F* = 6.946***

*Note*: The number in parentheses is the sample size. “SD” indicates standard deviation. The “Balance” column shows analysis of variance (ANOVA) *F* values.

**p* < 0.05,

***p* < 0.01.

****p* < 0.001.
