# Supplementary material for: Virtual Flood Experience Enhances Preparedness Through Emotional and Neural Engagement
Source: Risk Anal. 2026 Nov 4;46(11):e70407. doi: 10.1111/risa.70407 (PMC13734289; doi:10.1111/risa.70407)
Supplement: Supplementary file 1 [file RISA-46-0-s001.pdf]

Supplementary Information

Virtual flood experience enhances preparedness through emotional and neural engagement

This PDF file includes:

Table S1. Simultaneous estimation results from the individual fixed-effects panel mediation model

Table S2. Full estimation results from the simultaneous individual fixed-effects panel mediation model using the six-action preparedness-intention scale

Table S3. Direct and mediator-specific total indirect effects from the simultaneous individual fixed-effects panel mediation model using the six-action preparedness-intention scale

Table S4. Full estimation results from the ANCOVA-based simultaneous path model

Table S5. Direct and mediator-specific total indirect effects from the ANCOVA-based simultaneous path model

Figure S1. Changes in flood preparedness intention using the six-action preparedness-intention scale

Table S1. Simultaneous estimation results from the individual fixed-effects panel mediation model

Variable	Fear	Worry	Risk perception (frequency)	Risk perception (magnitude)	Personal relevance	Intention
Flood	0.421*** (0.062)	0.198* (0.096)	0.292** (0.103)	0.334** (0.102)	0.264* (0.103)	0.004 (0.074)
Video	-0.276*** (0.063)	0.190* (0.084)	0.245** (0.091)	0.297** (0.092)	0.271** (0.100)	-0.036 (0.067)
Flood×Video	1.149*** (0.088)	-0.370** (0.122)	-0.517*** (0.138)	-0.625*** (0.140)	-0.487*** (0.143)	0.057 (0.102)
Fear		0.328*** (0.049)	0.403*** (0.051)	0.331*** (0.048)	0.320*** (0.052)	0.155*** (0.040)
Worry						0.128** (0.047)
Risk perception (frequency)						0.057 (0.032)
Risk perception (magnitude)						0.077* (0.035)
Personal Relevance						0.146*** (0.036)

Note. This table reports unstandardized coefficients from the simultaneous individual fixed-effects panel mediation model used in the main analysis. The analysis included 1,000 participants in the web experiment: flood videos, n = 249; flood maps, n = 256; normal videos, n = 246; and normal maps, n = 249. Each column represents an outcome equation for fear, worry, risk perception of frequency, risk perception of magnitude, personal relevance, or flood preparedness intention. Standard errors are shown in parentheses. Global model-fit indices are not reported because the path model was just-identified/saturated and such indices are therefore not informative. *p < .05, **p < .01, ***p < .001.

Table S2. Full estimation results from the simultaneous individual fixed-effects panel mediation model using the six-action preparedness-intention scale

Variable	Fear	Worry	Risk perception (frequency)	Risk perception (magnitude)	Personal relevance	Intention
Flood	0.421***<br>(0.062)	0.198*<br>(0.096)	0.292**<br>(0.103)	0.334**<br>(0.102)	0.264*<br>(0.103)	0.008<br>(0.067)
Video	-0.276***<br>(0.063)	0.190*<br>(0.084)	0.245**<br>(0.091)	0.297**<br>(0.092)	0.271**<br>(0.100)	-0.009<br>(0.060)
Flood×Video	1.149***<br>(0.088)	-0.370**<br>(0.122)	-0.517***<br>(0.138)	-0.625***<br>(0.140)	-0.487***<br>(0.143)	-0.001<br>(0.091)
Fear		0.328***<br>(0.049)	0.403***<br>(0.051)	0.331***<br>(0.048)	0.320***<br>(0.052)	0.140***<br>(0.034)
Worry						0.145***<br>(0.040)
Risk perception (frequency)						0.047<br>(0.033)
Risk perception (magnitude)						0.079*<br>(0.031)
Personal Relevance						0.157***<br>(0.033)

Note. This table reports unstandardized coefficients from the simultaneously estimated individual fixed-effects panel mediation model using the six-action version of the flood preparedness-intention scale. This supplementary analysis used the same model specification as the main analysis, except that preparedness intention was calculated as the mean of six preparedness actions: making an evacuation plan, stockpiling drinking water, food, and daily necessities, preparing an emergency bag, purchasing flood insurance, checking a flood risk map of the residential area, and checking flood warning maps in the event of flood danger. The analysis included 1,000 participants in the web experiment: flood videos, $n = 249$; flood maps, $n = 256$; normal videos, $n = 246$; and normal maps, $n = 249$. Each column represents an outcome equation for fear, worry, risk perception of frequency, risk perception of magnitude, personal relevance, or flood preparedness intention. Individual fixed effects were included to account for time-invariant individual heterogeneity. Standard errors are shown in parentheses. Global model-fit indices are not reported because the path model was just-identified/saturated and such indices are therefore not informative. *$p < .05$, **$p < .01$, ***$p < .001$.

Table S3. Direct and mediator-specific total indirect effects from the simultaneous individual fixed-effects panel mediation model using the six-action preparedness-intention scale

Mediation pathways	Estimate	SE	95% CI	P-value	Holm-adjusted p
Flood×Video → (direct) → Intention	-0.001	0.091	[-0.175, 0.178]	0.994	–
Flood×Video → Fear → Intention	0.325	0.053	[0.226, 0.434]	<0.001	<0.001
Flood×Video → Worry → Intention	0.001	0.018	[-0.034, 0.041]	0.959	1.000
Flood×Video → Risk perception (frequency) → Intention	-0.003	0.008	[-0.022, 0.012]	0.763	1.000
Flood×Video → Risk perception (magnitude) → Intention	-0.019	0.013	[-0.051, 0.001]	0.148	0.592
Flood×Video → Personal relevance → Intention	-0.019	0.021	[-0.060, 0.024]	0.377	1.000

Note. Estimates are based on the simultaneous individual fixed-effects panel mediation model using the six-action version of the flood preparedness-intention scale. The six-action scale includes the four preparedness actions used in the main analysis plus two map-related actions: checking a flood risk map of the residential area and checking flood warning maps in the event of flood danger. The analysis included 1,000 participants in the web experiment: flood videos, $n = 249$; flood maps, $n = 256$; normal videos, $n = 246$; and normal maps, $n = 249$. Estimates represent the direct effect and mediator-specific total indirect effects of the Flood × Video interaction on preparedness intention. Flood × Video denotes the interaction between flood versus normal content and video versus map format. For worry, risk perception of frequency, risk perception of magnitude, and personal relevance, the estimates include indirect pathways in which fear is an upstream mediator. These effects are therefore not mutually exclusive and should not be summed. Standard errors, percentile-based 95% confidence intervals, and p-values were obtained from 5,000 bootstrap resamples. Holm-adjusted *p*-values were calculated across the five mediator-specific total indirect effects, excluding the direct effect.

Table S4. Full estimation results from the ANCOVA-based simultaneous path model

Variable	Fear	Post_Worry	Post_Risk perception (frequency)	Post_Risk perception (magnitude)	Post_Personal relevance	Post_Intention
Flood	0.421*** (0.062)	0.038 (0.078)	0.198* (0.081)	0.156 (0.079)	0.009 (0.082)	-0.073 (0.060)
Video	-0.276*** (0.063)	0.224** (0.079)	0.268** (0.084)	0.285** (0.086)	0.218* (0.089)	0.020 (0.059)
Flood×Video	1.149*** (0.088)	-0.811*** (0.117)	-0.928*** (0.122)	-0.893*** (0.126)	-0.846*** (0.136)	-0.024 (0.090)
Fear		0.758*** (0.051)	0.739*** (0.047)	0.648*** (0.046)	0.786*** (0.054)	0.185*** (0.039)
Pre_Worry		0.367*** (0.032)				-0.109** (0.039)
Post_Worry						0.158** (0.047)
Pre_Risk perception (frequency)			0.307*** (0.029)			-0.037 (0.026)
Post_Risk perception (frequency)						0.040 (0.038)
Pre_Risk perception (magnitude)				0.342*** (0.333)		-0.043 (0.030)
Post_Risk perception (magnitude)						0.090* (0.038)
Pre_Personal Relevance					0.342*** (0.033)	-0.025 (0.033)
Post_Personal Relevance						0.273*** (0.040)
Pre_Intention						0.545*** (0.036)

Note. This table reports unstandardized coefficients from the ANCOVA-based simultaneous path model estimated as a robustness check for the main individual fixed-effects panel mediation model. The analysis included 1,000 participants in the web experiment: flood videos, $n = 249$; flood maps, $n = 256$; normal videos, $n = 246$; and normal maps, $n = 249$. Each column represents an outcome equation for fear, post-stimulus worry, post-stimulus risk perception of frequency, post-stimulus risk perception of magnitude, post-stimulus personal relevance, or post-stimulus flood preparedness intention. The path structure was the same as that used in the main mediation analysis, except that post-stimulus outcomes were adjusted for their corresponding pre-stimulus values, rather than estimated using individual fixed effects. Fear was measured after each stimulus and therefore had no pre-stimulus covariate. Standard errors are shown in parentheses. *$p < .05$, **$p < .01$, ***$p < .001$.

Table S5. Direct and mediator-specific total indirect effects from the ANCOVA-based simultaneous path model

Mediation pathways	Estimate	SE	95% CI	P-value	Holm-adjusted p
Flood×Video → (direct) → Intention	-0.024	0.091	[-0.197, 0.154]	0.789	–
Flood×Video → Fear → Intention	0.697	0.084	[0.534, 0.866]	<0.001	<0.001
Flood×Video → Worry → Intention	0.009	0.021	[-0.031,0.055]	0.657	1.000
Flood×Video → Risk perception (frequency) → Intention	-0.003	0.008	[-0.023, 0.011]	0.698	1.000
Flood×Video → Risk perception (magnitude) → Intention	-0.013	0.014	[-0.047, 0.008]	0.337	1.000
Flood×Video → Personal relevance → Intention	0.015	0.038	[-0.058, 0.088]	0.697	1.000

Note. Estimates are based on the ANCOVA-based simultaneous path model estimated as a robustness check for the main individual fixed-effects panel mediation model. The analysis included 1,000 participants in the web experiment: flood videos, *n* = 249; flood maps, *n* = 256; normal videos, *n* = 246; and normal maps, *n* = 249. Estimates represent the direct effect and mediator-specific total indirect effects of the Flood × Video interaction on post-stimulus flood preparedness intention. The ANCOVA-based model adjusted post-stimulus outcomes for their corresponding pre-stimulus values where applicable; fear was measured after each stimulus and therefore had no pre-stimulus covariate. Flood × Video denotes the interaction between flood versus normal content and video versus map format. For worry, risk perception of frequency, risk perception of magnitude, and personal relevance, the estimates include indirect pathways in which fear is an upstream mediator. These effects are therefore not mutually exclusive and should not be summed. Standard errors, percentile-based 95% confidence intervals, and p-values were obtained from 5,000 bootstrap resamples. Holm-adjusted *p*-values were calculated across the five mediator-specific total indirect effects, excluding the direct effect.

Figure S1. Changes in flood preparedness intention using the six-action preparedness-intention scale

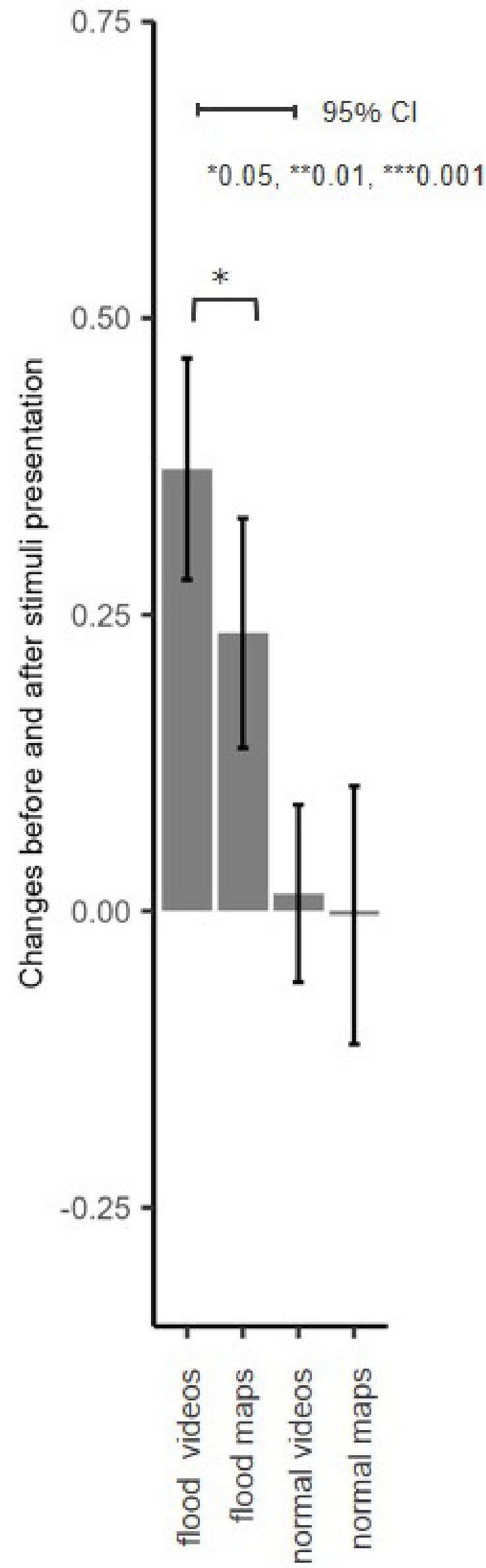

Note. Bars indicate mean changes in flood preparedness intention from before to after stimulus exposure for each experimental condition: flood videos, flood maps, normal videos, and normal maps. In this supplementary analysis, preparedness intention was calculated as the mean of six preparedness actions, including the four actions used in the main analysis plus two map-related actions: checking a flood risk map of the residential area and checking flood warning maps in the event of flood danger. The analysis included 1,000 participants: flood videos, $n = 249$; flood maps, $n = 256$; normal videos, $n = 246$; and normal maps, $n = 249$. Error bars indicate 95% confidence intervals. The bracket indicates the two-sample t-test comparing changes between the flood video and flood map conditions. *$p < .05$, **$p < .01$, ***$p < .001$.
